# Prediction of Gastric Cancer-Related Proteins Based on Graph Fusion Method

**DOI:** 10.3389/fcell.2021.739715

**Published:** 2021-11-01

**Authors:** Hao Zhang, Ruisi Xu, Meng Ding, Ying Zhang

**Affiliations:** Endoscopy Center, China-Japan Union Hospital of Jilin University, Changchun, China

**Keywords:** gastric cancer, protein, proteomics data, graph convolutional network, Xgboost

## Abstract

Gastric cancer is a common malignant tumor of the digestive system with no specific symptoms. Due to the limited knowledge of pathogenesis, patients are usually diagnosed in advanced stage and do not have effective treatment methods. Proteome has unique tissue and time specificity and can reflect the influence of external factors that has become a potential biomarker for early diagnosis. Therefore, discovering gastric cancer-related proteins could greatly help researchers design drugs and develop an early diagnosis kit. However, identifying gastric cancer-related proteins by biological experiments is time- and money-consuming. With the high speed increase of data, it has become a hot issue to mine the knowledge of proteomics data on a large scale through computational methods. Based on the hypothesis that the stronger the association between the two proteins, the more likely they are to be associated with the same disease, in this paper, we constructed both disease similarity network and protein interaction network. Then, Graph Convolutional Networks (GCN) was applied to extract topological features of these networks. Finally, Xgboost was used to identify the relationship between proteins and gastric cancer. Results of 10-cross validation experiments show high area under the curve (AUC) (0.85) and area under the precision recall (AUPR) curve (0.76) of our method, which proves the effectiveness of our method.

## Introduction

Gastric cancer is a worldwide disease with high incidence rate and mortality rate, especially in East Asia (Villanueva, 2011). According to the data from GLOBOCAN in 2018, there were 1,033,701 new cases of and 782,685 deaths from gastric cancer in the world (Bray et al., 2018). At present, the early diagnosis of gastric cancer is limited; patients are usually diagnosed in advanced stage. Therefore, early diagnosis is the key to improve the prognosis of patients, which is also the goal pursued by many researchers (Jin et al., 2015). Biomarkers refer to the substances that can reflect the physiological, biochemical, immune, genetic, and other molecular changes in the organism (Szász et al., 2016; Liang et al., 2019; Cheng et al., 2021). The levels of biomarkers in patients’ samples (such as blood, plasma, saliva, and urine) can reflect the health or disease status of patients, as well as the response to anticancer treatment. Due to the strong heterogeneity of gastric cancer, the use of proteomics technology to find new specific biomarkers will greatly improve the sensitivity and accuracy of the diagnosis of patients (Gullo et al., 2018).

Although many researchers tend to reveal diseases pathogenic mechanism by genomics (Peng and Zhao, 2020; Zhao et al., 2020b; Zhou et al., 2020), changes in protein quality in diseases reflect the progression of the disease and are also the product of genes (Zhao et al., 2020c). Unlike those studies that research diseases through gene expression (Zhao et al., 2021b), protein quantification is more accurate and has the potential to become a biomarker. Researchers have used various protein separation techniques, such as two-dimensional gel electrophoresis (2-DE) (Gygi et al., 2000), two-dimensional fluorescence difference gel electrophoresis (2D-DIGE) (Tannu and Hemby, 2006), isobaric tags for relative and absolute quantitation (iTRAQ), hydrophilic interaction liquid chromatography (HILIC) screening of potential target proteins of new gastric cancer biomarkers, and then Western blotting and enzyme-linked immunosorbent assay or immunohistochemistry (IHC) methods are further validated, and biomarkers that play a key role in the occurrence of malignant tumors can be discovered.

Ryu et al. (2003) used the tumor proteomics technology of antibody microarrays to identify inflammatory protein markers of gastric cancer. They found that 14 proteins have different expressions between normal gastric mucosa and tumor gastric mucosa. The proteome can be regarded as the functional cell equivalent of the genome. Proteomics is useful in discovering biomarkers and improving the diagnostic efficiency of early gastric cancer and has obvious advantages. At present, the prognosis and treatment methods of gastric cancer are guided by genome. Surgical resection is still the most common strategy of gastric cancer, but due to the high risk of disease progression in stage II or III patients, it becomes important to increase adjuvant therapy. The strong heterogeneity of gastric cancer makes the therapeutic effect heterogeneous. Therefore, although the TNM system can help the prognosis of gastric cancer, many researchers tend to discover biomarkers to predict treatment outcomes more accurately (Pang et al., 2018). For example, Balluff et al. (2011) used matrix-assisted laser desorption/ionization (MALDI) imaging technology to analyze tissue samples and found that cysteine-rich intestinal protein 1 (CRIP1) and human neutrophil peptide-1 (HNP-1) were prognostic factors for gastric cancer. Human epidermal growth factor receptor 2 (HER2) is an important biomarker in gastric tumors, which can be specifically targeted for treatment with trastuzumab monoclonal antibody (mAb). For patients with advanced gastric cancer or gastroesophageal junction cancer, trastuzumab combined with chemotherapy can improve the survival rate of patients (Park et al., 2018).

There are still few proteins known to be related to gastric cancer. With the explosive growth of various types of omics data (Mo et al., 2020; Zhao et al., 2020a,2021a), computational methods are widely used to identify disease-related biomolecules. Mining disease-related molecules based on the protein interaction networks has become a universal method. Sang et al. (2011) discovered genes and pathways of ciliopathy disease based on protein network. Seyfried et al. (2017) constructed protein network to identify protein-specific co-expression in Alzheimer’s disease. With the development of Graph Convolutional Networks (GCN), an increasing number of researchers tend to use this method to process the complex topological features of the biological network. Its core point of view is to make the entire graph converge through the dissemination of node information and then make predictions on the basis of it. It has been widely used in prediction of biomolecular interaction (Tianyi et al., 2020). Therefore, we proposed a GCN-based method in this paper, named “GXGCP” (Gcn-Xgboost for Gastric Cancer-related Proteins identification) to identify gastric cancer-related proteins.

## Materials and Methods

**FIGURE 1:**
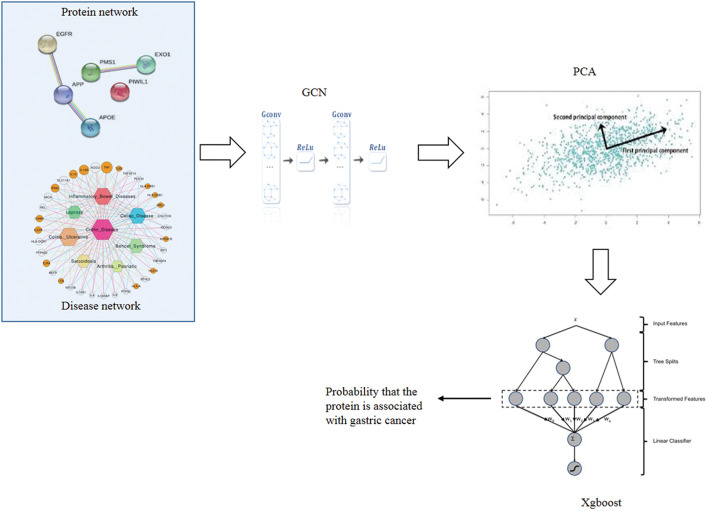
Workflow of GXGCP (Gcn-Xgboost for Gastric Cancer-related Proteins identification).

There are four steps to implement GXGCP. Step 1 is to construct disease similarity network and protein interaction network. Step 2 is using GCN to extract topological features of disease similarity network and protein interaction network, respectively. Step 3 is to reduce the dimension of protein and gastric cancer features by principal component analysis (PCA). Step 4 is to identify gastric cancer-related proteins based on the features of protein and gastric cancer by Xgboost. The workflow of GXGCP is shown in Figure 1.

### Construction of Network

We used SemFunsim (Cheng et al., 2014) to obtain diseases that are similar to gastric cancer. This method considers both disease semantic association and gene association. The detailed calculation process will not be repeated in this paper. A total of 327 diseases were found to be similar to gastric cancer. Based on the similarity, we constructed disease network, in which the edges are similarity and nodes are diseases. Therefore, the network has weight.

We downloaded protein interaction information from Search Tool for the Retrieval of Interacting Genes/Proteins (STRING) (Mering et al., 2003). Based on the interaction, we constructed a protein network. If a protein can interact with the other one, there would be an edge to connect each other. Since the intensity of interaction between different proteins is different, this network also has weight.

### Extracting Topological Features by Graph Convolutional Networks

To fully extract topological features of protein and disease network, GCN was applied (Han et al., 2019). The aim to implement GCN is to convert network topology into a vector output:


(1) $$H^{\left(l+1\right)}=G\,C\,N\,\left(H^{\left(l\right)},A\right)$$

where *H*^(0)^ is node’s feature in the network.

First, Laplace transform should be done on the network:


(2) L=D-A

where D is the degree matrix of the network, and A is the adjacency matrix.


(3) $${\hat{\text{D}}}_{\text{ii}}=\sum_{j}{\hat{\text{A}}}_{i\,j}$$

Then, normalization should be implemented on the Laplacian matrix:


(4) $$L^{s\,y\,m}=D^{-\frac{1}{2}}\,L\,D^{-\frac{1}{2}}=I-D^{-\frac{1}{2}}\,A\,D^{-\frac{1}{2}}$$

*L*^*sym*^ is defined as:


(5) $$L_{i,j}^{s\,y\,m}=\left\{ \begin{array}{c} 1\;i=j\,a\,n\,d\,\deg\left(v_{i}\right)\neq 0\\ -\frac{1}{\sqrt{\deg\left(v_{i}\right)\,\deg\left(v_{j}\right)}}\;i\neq j\,a\,n\,d\,v_{i}\,a\,d\,j\,a\,c\,e\,n\,t\,t\,o\,v_{j}\\ 0\;o\,t\,h\,e\,r\,w\,i\,s\,e \end{array}\right.$$

With the Laplacian matrix, we can perform spectral convolution on the network. We need to find a suitable convolution kernel so that *f*() can reduce the loss of classification after the convolution transformation of the convolution kernel. The core of the machine learning task on the graph is to find a convolution kernel that can reduce the loss, regard *h*(λ_1_),…*h*(λ_*n*_) as the parameters of the model, and apply the gradient descent method to update these parameters.

The final formula of GCN would be:


(6) $$H^{\left(l+1\right)}=\sigma \,\left(D^{-\frac{1}{2}}\,A\,D^{-\frac{1}{2}}\,H^{\left(l\right)}\,W^{\left(l\right)}\right)$$

where σ() is the activation function, and *W*^(*l*)^ is the parameter to be trained.

### Reduction Dimension by Principal Component Analysis

Since the dimension of metabolites and gastric cancer features are large, we used PCA to reduce the dimension. There are four steps to apply PCA (Tipping and Bishop, 1999; Cheng et al., 2019). The first step is feature centralization. That is, the data of each dimension are subtracted from the mean value of that dimension, and the mean value of each dimension becomes 0 after the transformation. The second step is to calculate covariance matrix. The third step is to calculate the eigenvalues and eigenvectors of the covariance matrix. The last step is to select the feature vector corresponding to the large feature value to obtain a new data set.

### Classification of Gastric Cancer-Related Proteins by Xgboost

Xgboost is a sparse perception algorithm that can be used for parallel tree learning (Chen and Guestrin, 2016). Since the features of gastric cancer and proteins are sparse, Xgboost is very suitable for the classification.

Xgboost is a tree ensemble model. It sums the results of K (the number of trees) as the final predicted value.


(7) $${\overset{⌢}{y}}_{i}=\phi \left(x_{i}\right)=\sum_{k=1}^{K}f_{k}\left(x_{i}\right),f_{k}\in F$$

Assuming that a given sample set has n samples and m features, then


(8) $$D=\left\{\left(x_{i},y_{i}\right)\right\}$$

where *x*_*i*_ represents the i-th sample, *y*_*i*_ represents the i-th category label, and the space F of the regression tree (CART tree) is:


(9) $$F=\left\{f\left(x\right)=w_{q}\left(x\right)\right\}$$

where q represents the structure of each tree, it maps the sample to the corresponding leaf node; T is the number of leaf nodes of the corresponding tree; f(x) corresponds to the structure q of the tree and the leaf node weight w. Therefore, the predicted value of Xgboost is the sum of the values of the leaf nodes corresponding to each tree.

Our goal is to learn these k trees, so we minimize the following objective function with regular terms:


(10) $$L\,\left(\phi \right)=\sum_{i}l\,\left({\overset{⌢}{y}}_{i},y_{i}\right)+\sum_{k}\Omega \,\left(f_{k}\right)$$

where $\Omega \,\left(f\right)=\gamma \,T+\frac{1}{2}\,\lambda \,{||w||}^{2}$

## Experiment Results

We implemented 10-cross validation experiments to test the performance of GXGCP. We divided our data into 10 groups. We used nine of 10 groups’ data to train the model and the data of the remaining one to test the model. After repeating this process 10 times, each group has been tested once. To show the accuracy of our model, we compared GXGCP with several other methods such as RWXGCP, GXGCP without PCA, GSVMCP, GANNCP, and GCNNCP. RWXGCP replaces the GCN part of GXGCP with random walk (RW). GSVMCP replaces the Xgboost part of GXGCP with support vector machine (SVM). GANNCP replaces the Xgboost part of GXGCP with artificial neural network (ANN). GCNNCP replaces the Xgboost part of GXGCP with convolutional neural network (CNN).

**FIGURE 2:**
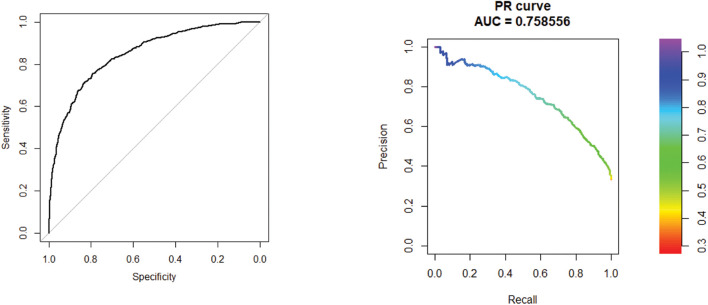
Receiver operating characteristic (ROC) curve of 10-cross validation experiments. The AUC and AUPR curve of GXGCP are 0.85 and 0.76, respectively. The comparison results are listed in Table 1.

The area under the curve (AUC) and area under the precision recall (AUPR) curve of GXGCP are shown in Figure 2. The comparison results are listed in Table 1.

**TABLE 1:** Comparison results.

Method	AUC	AUPR
GXGCP	0.85	0.76
RWXGCP	0.81	0.72
GXGCP without PCA	0.72	0.68
GSVMCP	0.74	0.65
GANNCP	0.76	0.71
GCNNCP	0.82	0.76
GDNNCP	0.80	0.74

As shown in Table 1, GXGCP performed best among these five methods. These results show that GCN is more suitable for encoding network than RW, and Xgboost is more suitable for building model by sparse data than SVM and ANN.

## Conclusion

Protein is the main executor of life activities. To decrypt the genome, you must first systematically understand the proteome. Identifying gastric cancer-related proteins can greatly help develop screening or testing tools for tumor detection, early diagnosis or differential diagnosis, prognostic analysis, efficacy evaluation, etc. Due to the high cost of biological experiments, we proposed GXGCP that fuses GCN, Xgboost, and PCA to identify gastric cancer-related proteins. To verify the accuracy of our method, we did 10-cross validation experiments. The results show that the AUC of GXGCP reached 0.85 and AUPR reached 0.76. To show the superiority of GXGCP, we compared it with several other methods, and GXGCP performed best. Overall, we propose a novel, efficient, and accurate method for large-scale identification of gastric cancer-related proteins, which would greatly benefit the study of the pathogenic mechanism and clinical research of gastric cancer.

## Data Availability Statement

The datasets presented in this study can be found in online repositories. The names of the repository/repositories and accession number(s) can be found in the article/supplementary material.

## Ethics Statement

Ethical review and approval was not required for the study on human participants in accordance with the local legislation and institutional requirements. Written informed consent for participation was not required for this study in accordance with the national legislation and the institutional requirements.

## Author Contributions

HZ and RX wrote this manuscript and did experiments. MD and YZ provided important ideas. All authors read and approved the final manuscript.

## Conflict of Interest

The authors declare that the research was conducted in the absence of any commercial or financial relationships that could be construed as a potential conflict of interest.

## Publisher’s Note

All claims expressed in this article are solely those of the authors and do not necessarily represent those of their affiliated organizations, or those of the publisher, the editors and the reviewers. Any product that may be evaluated in this article, or claim that may be made by its manufacturer, is not guaranteed or endorsed by the publisher.
